# Inequalities in short-acting reversible, long-acting reversible and permanent contraception use among currently married women in India

**DOI:** 10.1186/s12889-022-13662-3

**Published:** 2022-06-28

**Authors:** Milan Das, Abhishek Anand, Babul Hossain, Salmaan Ansari

**Affiliations:** 1 Department of the Population and Development, International Institute for Population Sciences (IIPS), Mumbai, India; 2 Department of Family and Generations, International Institute for Population Sciences (IIPS), Mumbai, India; 3 International Institute for Population Sciences (IIPS), Mumbai, India; 4 Department of Population Policies and Programs, International Institute for Population Sciences (IIPS), Mumbai, India

**Keywords:** Inequality, Currently married women, Family planning, Modern contraceptive, National Family Health Survey, India

## Abstract

**Background:**

In India, the usage of modern contraception methods among women is relatively lower in comparison to other developed economies. Even within India, there is a state-wise variation in family planning use that leads to unintended pregnancies. Significantly less evidence is available regarding the determinants of modern contraception use and the level of inequalities associated with this. Therefore, the present study has examined the level of inequalities in modern contraception use among currently married women in India.

**Methods:**

This study used the fourth round of National Family Health Survey (NFHS-4) conducted in 2015-16. Our analysis has divided the uses of contraception into three modern methods of family planning such as Short-Acting Reversible Contraception (SARC), Long-Acting Reversible Contraception (LARC) and permanent contraception methods. SARC includes pills, injectable, and condoms, while LARC includes intrauterine devices, implants, and permanent contraception methods (i.e., male and female sterilization). We have employed a concentration index to examine the level of socioeconomic inequalities in utilizing modern contraception methods.

**Results:**

Our results show that utilization of permanent methods of contraception is more among the currently married women in the higher age group (40–49) as compared to the lower age group (25–29). Women aged 25–29 years are 3.41 times (OR: 3.41; 95% CI: 3.30–3.54) more likely to use SARC methods in India. Similarly, women with 15 + years of education and rich are more likely to use the LARC methods. At the regional level, we have found that southern region states are three times more likely to use permanent methods of contraception. Our decomposition results show that women age group (40–49), women having 2–3 children and richer wealth quintiles are more contributed for the inequality in modern contraceptive use among women.

**Conclusions:**

The use of SARC and LARC methods by women who are marginalized and of lower socioeconomic status is remarkably low. Universal free access to family planning methods among marginalized women and awareness campaigns in the rural areas could be a potential policy prescription to reduce the inequalities of contraceptive use among currently married women in India.

## Introduction

Contraceptive use is one of the proximate determinants of fertility and a major predictor of fertility transition and family planning in developing countries. According to theoretical framework outlined by Bongaart (1978), one of the factors influencing the overall change in fertility at the population level is the change in the prevalence of contraception, which operates as an intermediate fertility variable [1, 2]. Further, the level of contraception use reflects the societies' attitudes and behaviours towards women and women's autonomy in the community [3, 4]. The prevalence of contraception use also reveals gender equality and the quality of public health programs [5–7]. As a result, studies on contraceptive use have depicted the effects of contraception on demographic transition and population development [2, 5–7].

Literature shows there are various factors such as limited access to contraceptives, fear of side effects from modern contraceptives, social norms, and cultural and religious beliefs which contribute to the inequity in the use of modern contraceptives in developing countries [8]. Therefore, developing countries adopted a mix of contraception methods that includes Short-Acting Reversible Contraception (SARC^1^), Long-Acting Reversible Contraception (LARC) and permanent contraception, which can probably be an alternative family planning strategy to meet the high unmet need for modern contraception [9]. Previous research suggested that providing a wide range of mixed contraceptive methods might increase the contraceptive prevalence and lead to better family planning [10, 11]. Studies in developing countries indicated that countries with more access to different methods of contraception (i.e. SARC, LARC and permanent contraception) have led to higher contraception prevalence [12, 13]. However, SARC methods are the most common, while LARC methods are more cost-effective than SARC [14, 15]. At the same time, permanent contraceptive methods are preferred for their convenience, lack of side effects and ease of use but are often associated with invasive procedures [16].

Even though there are pros and cons to each group of modern contraceptive methods, studies have revealed a significant regional differences and inequalities in the adoption of mixed contraceptive methods [17, 18]. SARC methods are more common in Africa and Europe than other methods, while LARC or permanent contraceptive methods are more common in Asia and Northern America [17]. According to Sullivan and colleagues (2005), women in developing countries such as India, Dominican Republic, Brazil, and Panama are more likely to use female sterilization. While, the Sub-Saharan African countries and Norther/West African counties are predominately used traditional methods of contraception such as the SARC method [18]. But previous literature indicates significant social-economic inequalities exist among women that generate a usage gap of different methods of contraception. Socioeconomic inequalities exist among communities in terms of education, social, and wealth status [19]. A study conducted by Ugaz and colleagues (2016) found that wealthy women are more likely to practice LARC and permanent contraceptive methods than the SARC methods, and SARC is the most preferred method of contraception among the poorer women [20].

As per the National Family Health Survey (NFHS) data, the prevalence of any method of contraception has increased significantly from 40.7% in 1992–93 to 53.3% in 2015–16 in India. While the adoption of any modern method of contraception has increased from 36.5% in 1992–93 to 47.8% in 2015–16. On the contrary, high levels of variation in the mix of modern contraception methods were reported in the literature that might be due to various socioeconomic differences among women's households and regional level factors [21, 22]. In India, the majority of studies have focused on the selection and use of family planning methods, unmet needs, and demand for family planning [21, 23, 24]. Another set of studies has investigated the changes in the method of contraception and identified factors associated with contraceptive use in India [21, 22]. However, there are no studies that have explored the level of inequality in the usage of mixed methods of contraception among currently married women in India. Therefore, this study has measured socioeconomic inequality of different methods of contraception (i.e. SARC, LARC, and permanent contraception methods) using concentration curve and concentration index. This study has used a currently married women sample, which is unique as compared to past studies because it is evidenced that most births in India occur within unions and births outside the union are not socially acceptable. As a result, this research is extremely important in light of India's recent fertility decline.

## Methodology

### Source of data

The study used the nationally representative National Family Health Survey (2015–16) data in India. The NFHS is conducted in line with the Global Demographic and Health Survey (DHS).The NFHS is a cross-sectional survey conducted under the stewardship of the Ministry of Health and Family Welfare (MoHFW) (ICF, IIPS, 2017). NFHS used a two-stage stratified sampling method, and they came from all 36 states of India and union territories. The sampling techniques and procedures are mentioned elsewhere. The main objective of NFHS is to provide various estimate indicators such as maternal and child health, fertility, mortality, nutrition, family planning, domestic violence, and women empowerment. Around 699,686 women in the reproductive age groups (15–49) were interviewed from 601,509 households samples from India’s states and union territories. This study aims to analyse inequality in contraceptive use among Indian women. Hence, we restricted our analysis to currently married women aged 15–49. The final analytical sample size was 499,687 currently married women.

### Outcome variables

Our primary outcome variable for the study was the types of contraceptive use for the analysis. In this study used three types of modern contraception methods such as Short-Acting Reversible Contraceptives (SARC), which contained condoms, oral contraceptives, pills, injectable hormones and, spermicide; Long-Acting Reversible Contraceptives (LARC) which included intrauterine devices (IUD) and implants; and Permanent Contraception Methods, including male and female sterilization [25]. Therefore, women responding to their current contraceptive methods are above the list of the different contraceptive methods, which is further coded as binary variables. For instance, if women used SARC methods, coded as ‘1’ and the not used ‘0’. Women used the LARC methods coded as the ‘1’ and not used ‘0’. And if women used the permanent contraception methods coded as ‘1’ and not used ‘0’.

### Predictor variable

A thorough literature review was done, and control variables were considered because of their statistically significant relationship with contraceptive use (REF) [5]. These variables included the respondent age (15–19, 20–24, 25–29, 30–34, 35–39 and 40–49); total children ever born (0, 1, 2–3, and 4 +); women’s years of education (no education, 1–5, 6–9, 10–11, 12–14, and 15 + years); place of residence (urban and rural); religion (Hindu, Muslim, Others religion, ‘other’ religion included Christian, Sikh, Buddhist, Jain and other); caste ( Schedule Caste and Tribes, Other Backward Class (OBC), Others and ‘others’ caste included general category); and geographical regions included 28 states and 7 Union Territories (UT) (The north region included Jammu & Kashmir, Himachal Pradesh, Punjab, Rajasthan, Chandigarh, Uttarakhand, Haryana and Delhi; central region included: Uttar Pradesh, Chhattisgarh and Madhya Pradesh; east region included West Bengal, Jharkhand, Odisha, and Bihar; northeast region included Sikkim, Arunachal Pradesh, Nagaland, Manipur, Mizoram, Tripura, Meghalaya and Assam; the west region included Gujarat, Maharashtra, Goa, Dadra & Nagar Haveli and Daman and Diu, and finally south region included Andhra Pradesh, Karnataka, Kerala, Tamil Nadu, Puducherry, Telangana Lakshadweep and Andaman & Nicobar Islands); The NFHS-4 measured the economic status of household using wealth index scores assigned to each household assets, ownership of durable goods and access to various amenities. The survey used principal component analysis was used to create a composite variable of wealth index, which was coded as (poorer, poor, middle, richer and richest).

### Statistical analysis

Descriptive statistics and bivariate analysis were obtained to know the distribution and prevalence of the contraception methods and the Chi-square test was used to examine the relationship between socio-demographic characteristics and the use of contraceptive methods. Further, in the first stage, we used logistic regression to explore the socioeconomic determinants of contraceptive methods. The adjusted Odds ratio with 95% Confidence Interval (CI) were estimated using binary logistic regression analysis.

The equation for logistic distribution is:

$${l}_{n}\left(\frac{\pi }{1-\pi }\right)=\alpha +{\beta }_{1}{X}_{1}+{\beta }_{2}{X}_{2}+{\beta }_{3}{X}_{3}+\dots +{\beta }_{n}{X}_{n}$$

where ${X}_{1},{X}_{2}{,X}_{3},\dots {X}_{n}$ are explanatory variables and ${\beta }_{1},{\beta }_{2}{,\beta }_{3},\dots {\beta }_{n}$ are regression coefficients.

In the second stage, we also used concentration index and concentration curves to analyse the socioeconomic inequalities in contraceptive use. The equation of the contrition index and the decomposition of the concentration index is as follows.

## Concentration index

The concentration index and curve were used to determine the income-related inequalities in the use of short-acting reversible, long-acting reversible and permanent contraceptives. The mathematical expression of the concentration index is written as follows:

$$C=\frac{2}{\mu }cov({y}_{i},R)$$

where C is the concentration index, ${y}_{i}$ is outcome variables, and ***cov*** denotes covariance. The index varies between -1 to + 1, where the sign indicates the direction of the relationship, whereas magnitude shows the strength of the relationship. The zero value of the index implies that no inequality exists.

### Decomposition of the concentration index

The concentration index was further decomposed using Wagstaff decomposition to quantify the contribution of selected characteristics to the inequality in the use of short-acting reversible, long-acting reversible and permanent contraceptives. The Wagstaff decomposition technique is a regression-based approach to decomposing concentration index, and mathematically, it is depicted as:

$${t}_{i}=\alpha +\sum_{k=1}^{K}{\beta }_{k}{x}_{ik}+{\varepsilon }_{i}$$

where, ${y}_{i}$ is the variable of various contraceptive methods, ${x}_{ik}$ is the set of socioeconomic contributing factors and ${\varepsilon }_{i}$ is the error term. The concentration index can be rewritten as:

$$C=\sum \left(\frac{{\beta }_{k}{\overline{x} }_{k}}{\mu }\right){C}_{k}+\frac{GC\varepsilon }{\mu }/\mu$$

where $\mu$ denotes the mean of ${t}_{i}$, ${\overline{x} }_{k}$ is the mean of ${x}_{k}$, ${C}_{k}$ is the concentration index and $GC\varepsilon$ is the generalized concentration index for error ${(\varepsilon }_{i}).$ All things being constant, a positive (%) contribution by a factor would decrease socioeconomic inequality, whereas a negative (%) contribution would increase inequality (Mukong et al., 2017 [26]; Mutyambizi et al., 2019 [27]). The explained percentage contribution sums to 100 per cent, which depicts that the measured inequality is completely explained by selected predictor variables (Mondor et al., 2018 [28]).

All statistical analysis was performed using STATA 16.

## Results

### Socioeconomic and demographic characteristics

Table 1 shows the sample size distribution for SARC, LARC and Permanent contraception methods. Around 9.8% of women in the teenage age group used modern contraception. Almost 14% of the samples for the SARC methods were between the ages of 25–29. Only 2.2% of the women aged 25–29 used the LARC methods. However, 54% of women used permanent contraception in the age group 40–49. Approximately half of the women in the study used permanent contraception and had two to three children. Around 18% of the women with 15 + years of education used SARC methods. In addition, 43% of women used permanent contraception methods with no educational attainment. The SARC methods were used by 16% of women following in Muslim religion. In contrast, 36% of the women of other religions used permanent contraception methods. More than half of the women in the southern region used LARC methods.

**Table 1:** Sample characteristics by different modern contraceptive methods, 2015-16 (NFHS-4), India

Variables	Short Acting Reversible (SARC) (*n* = 49,370)	Long Acting Reversible (LARC) (*n* = 7,652)	Permanent Contraception (*n* = 181,170)	Total (*n* = 238,194)
	**n (%)**	**n (%)**	**n (%)**	**n (%)**
**Age**				
15–19	1,519 (8.4)	87 (0.5)	1,58 (0.9)	1,765 (9.8)
20–24	9,807 (12.5)	1,295 (1.7)	7,196 (9.2)	18,298 (23.3)
25–29	13,705 (13.7)	2,166 (2.2)	25,965 (25.9)	41,836 (41.7)
30–34	11,199 (12.6)	1,890 (2.1)	36,330 (40.9)	49,420 (55.6)
35–39	7,590 (9.2)	1,282 (1.6)	40,803 (49.6)	49,674 (60.4)
40–49	5,550 (4.2)	9,32 (0.7)	70,718 (53.7)	77,201 (58.6)
***p value***	< 0.001	< 0.001	< 0.001	< 0.001
**Children ever born**				
0	2,527 (5.1)	21 (0.04)	2,34 (0.5)	2,782 (5.6)
1	15,405 (16.9)	2,564 (2.8)	5,572 (6.1)	23,541 (25.8)
2–3	24,810 (9.5)	4,293 (1.6)	131,844 (50.4)	160,946 (61.5)
4 +	6,629 (6.8)	7,74 (0.8)	43,521 (44.8)	50,925 (52.4)
***p value***	< 0.001	< 0.001	< 0.001	< 0.001
**Women education**				
No Education	8,802 (5.3)	1,002 (0.6)	7,1241 (43.0)	81,046 (48.9)
1–5 years	6,220 (8.8)	7,35 (1.0)	30,342 (42.9)	37,296 (52.8)
6–9 years	13,282 (11.5)	1,887 (1.6)	41,019 (35.4)	56,188 (48.4)
10–11 years	6,958 (11.8)	1,198 (2.0)	19,700 (33.4)	27,857 (47.2)
12–14 years	6,305 (14.0)	1,269 (2.8)	10,550 (23.5)	18,125 (40.3)
15 +	7,803 (18.0)	1,562 (3.6)	8,318 (19.2)	17,683 (40.9)
***p value***	< 0.001	< 0.001	< 0.001	< 0.001
**Residence**				
Urban	21,397 (12.8)	3,951 (2.4)	60,036 (36.0)	85,383 (51.2)
Rural	27,974 (8.4)	3,702 (1.1)	121,135 (36.4)	152,812 (45.9)
***p value***	< 0.001	< 0.001	< 0.001	< 0.001
**Religion**				
Hindu	35,776 (8.8)	5,872 (1.4)	156,750 (38.5)	198,399 (48.8)
Muslim	10,233 (15.6)	9,23 (1.4)	13,751 (20.9)	24,907 (37.9)
Others	3,362 (12.5)	8,57 (3.2)	10,669 (39.6)	14,888 (55.2)
***p value***	< 0.001	< 0.001	< 0.001	< 0.001
**Caste**				
Schedule Caste and Tribes	12,167 (8.3)	1,813 (1.2)	56,197 (38.3)	70,177 (47.8)
Other Backward Caste (OBC)	16,368 (7.5)	3,053 (1.4)	81,813 (37.5)	101,235 (46.4)
Others	20,836 (15.5)	2,787 (2.1)	43,160 (32.0)	66,783 (49.6)
***p value***	< 0.001	< 0.001	p < 0.001	< 0.001
**Wealth**				
Poorer	5,787 (6.4)	4,91 (0.5)	26,625 (29.3)	32,904 (36.2)
Poor	8,994 (9.1)	8,96 (0.9)	35,130 (35.6)	45,021 (45.7)
Middle	8,849 (8.7)	1,073 (1.1)	41,384 (40.5)	51,307 (50.2)
Richer	10,464 (10.0)	1,909 (1.8)	41,958 (40.0)	54,331 (51.8)
Richest	15,276 (14.8)	3,283 (3.2)	36,073 (35.0)	54,632 (53)
***p value***	< 0.001	< 0.001	< 0.001	< 0.001
**Region**				
North	9,911 (15.7)	2,245 (3.6)	63,146 (36.3)	35,092 (55.6)
Central	12,515 (10.7)	1,223 (1.1)	116,739 (27.5)	45,799 (39.2)
East	15,394 (13.3)	1,031 (0.9)	115,524 (26.3)	46,752 (40.5)
Northeast	3,827 (22.6)	3,96 (2.3)	16,948 (9.9)	5,894 (34.8)
West	6,182 (8.6)	1,511 (2.1)	72,077 (45.1)	40,164 (55.7)
South	1,542 (1.3)	1,246 (1.1)	115,194 (53.6)	64,493 (56)
***p value***	< 0.001	< 0.001	< 0.001	< 0.001

### Likelihood of different modern contraceptive use by socio-demographic characteristics

Table 2 displays the adjusted odds of SARC, LARC, and Permanent contraception methods in India by socioeconomic characteristics. Women in the 40–49 age group were 12 times (OR:1.12;95% CI:1.10–1.14) more likely to use modern contraception. SARC methods were 3.41 times more likely to be used by women aged 25–29 years (OR:3.41;95% CI:3.30–3.54). Permanent methods of contraception were 0.07 times less likely to use in the 15–19 years age group (OR:0.07;95% CI:0.06–0.09) than in the reference category 40–49 years age group. SARC methods were 1.22 times (OR:1.22;95% CI:1.17–1.26) more likely to use in women with one child, LARC methods were 1.11 times more likely to use in women with one child, and permanent methods were 1.21 times more likely to use in women with two to three children than reference category women with four or more children. Women with a 15-year education were 2.88 times (OR:2.88; 95% CI:2.76–3.00) more likely to use SARC methods, 3.07 times (OR:3.07;95% CI:2.78–3.40) more likely to use LARC methods, and 0.40 times less likely to use permanent method contraception. Rural women were 1.13 times more likely to use permanent contraception than urban women. Richer women, on the other hand, were 2.20 times more likely to use SARC methods. Muslim women were 0.37 times less likely to use permanent contraception than Hindu women, and women of other religions were 0.96 times less likely to use it. Women from central region were 0.61 times less likely to use permanent contraception. And people in the south region were 3.00 times (OR:3.00, 95% CI:2.93–3.08) more likely to use permanent contraception.

**Table 2:** Logistic regression estimates of the short acting reversible, long acting reversible, and permanent contraception, 2015-16 (NFHS-4), India

Variables	Short Acting Reversible (SARC)		Long Acting Reversible (LARC)		Permanent Contraception		Total	
	**OR**	**95% CI**	**OR**	**95% CI**	**OR**	**95% CI**	**OR**	**95%CI**
***Age***								
15–19	3.24***	(3.03,3.47)	2.18***	(1.74,2.74)	0.07***	(0.06,0.09)	0.52***	(0.49,0.55)
20–24	3.32***	(3.19,3.45)	2.90***	(2.64,3.19)	0.20***	(0.19,0.20)	0.50***	(0.49,0.51)
25–29	3.41***	(3.30,3.54)	2.90***	(2.68,3.15)	0.39***	(0.38,0.40)	0.69***	(0.68,0.70)
30–34	3.12***	(3.01,3.23)	2.73***	(2.52,2.96)	0.66***	(0.65,0.68)	0.99	(0.98,1.01)
35–39	2.28***	(2.19,2.36)	2.09***	(1.92,2.28)	0.88***	(0.86,0.90)	1.12***	(1.10,1.14)
40–49®	Ref.		Ref.		Ref.		Ref.	
***Children Ever Born***								
0	0.30***	(0.28,0.31)	0.02***	(0.01,0.03)	0.01***	(0.01,0.01)	0.06***	(0.06,0.06)
1	1.22***	(1.17,1.26)	1.11*	(1.01,1.23)	0.10***	(0.10,0.10)	0.31***	(0.31,0.32)
2–3	0.97	(0.94,1.00)	0.95	(0.88,1.04)	1.21***	(1.19,1.24)	1.21***	(1.19,1.23)
4 + ®	Ref.		Ref.		Ref.		Ref.	
***Education***								
No Education®	Ref.		Ref.		Ref.		Ref.	
1–5 years	1.42***	(1.37,1.47)	1.37***	(1.24,1.51)	1.11***	(1.09,1.13)	1.18***	(1.16,1.20)
6–9 years	1.75***	(1.69,1.81)	1.84***	(1.69,2.00)	0.89***	(0.87,0.91)	1.08***	(1.06,1.10)
10–11 years	2.05***	(1.98,2.14)	2.06***	(1.88,2.27)	0.69***	(0.67,0.71)	0.94***	(0.91,0.96)
12–14 years	2.23***	(2.14,2.32)	2.61***	(2.37,2.88)	0.53***	(0.51,0.55)	0.86***	(0.84,0.89)
15 + years	2.88***	(2.76,3.00)	3.07***	(2.78,3.40)	0.40***	(0.38,0.41)	0.86***	(0.84,0.89)
***Residence***								
Urban®	Ref.		Ref.		Ref.		Ref.	
Rural	0.74***	(0.73,0.76)	0.76***	(0.73,0.81)	1.13***	(1.11,1.15)	0.99	(0.97,1.01)
***Wealth***								
Poorer®	Ref.		Ref.		Ref.		Ref.	
Poor	1.45***	(1.40,1.51)	1.34***	(1.20,1.50)	1.24***	(1.21,1.27)	1.45***	(1.42,1.48)
Middle	1.55***	(1.49,1.61)	1.33***	(1.19,1.49)	1.33***	(1.30,1.37)	1.62***	(1.59,1.66)
Richer	1.75***	(1.68,1.82)	1.88***	(1.68,2.11)	1.34***	(1.31,1.38)	1.74***	(1.70,1.78)
Richest	2.20***	(2.10,2.30)	2.33***	(2.07,2.63)	1.32***	(1.28,1.36)	1.93***	(1.88,1.98)
***Religion***								
Hindu®	Ref.		Ref.		Ref.		Ref.	
Muslim	1.78***	(1.73,1.83)	1.01	(0.94,1.09)	0.37***	(0.36,0.38)	0.60***	(0.59,0.61)
Others	1.21***	(1.16,1.26)	1.46***	(1.36,1.58)	0.96*	(0.93,0.99)	1.06***	(1.03,1.09)
***Caste***								
Schedule Caste and Tribes®	Ref.		Ref.		Ref.		Ref.	
Other backword calss	0.84***	(0.82,0.86)	1.05	(0.99,1.12)	0.93***	(0.91,0.94)	0.86***	(0.86,0.89)
Others	1.17***	(1.14,1.21)	1.02	(0.96,1.09)	0.94***	(0.92,0.96)	1.06***	(1.04,1.08)
***Region***								
North®	Ref.		Ref.		Ref.		Ref.	
Central	0.86***	(0.84,0.89)	0.40***	(0.37,0.43)	0.61***	(0.59,0.62)	0.55***	(0.53,0.56)
East	1.15***	(1.12,1.19)	0.39***	(0.36,0.42)	0.66***	(0.64,0.67)	0.67***	(0.66,0.69)
Northeast	1.82***	(1.73,1.90)	0.88*	(0.79,0.99)	0.18***	(0.17,0.19)	0.48***	(0.46,0.50)
West	0.46***	(0.45,0.48)	0.59***	(0.55,0.64)	1.77***	(1.73,1.82)	1.04***	(1.02,1.07)
South	0.07***	(0.06,0.07)	0.29***	(0.27,0.32)	3.00***	(2.93,3.08)	1.07***	(1.04,1.09)`

### Inequality in the different modern contraceptive uses

**Fig. 1:**
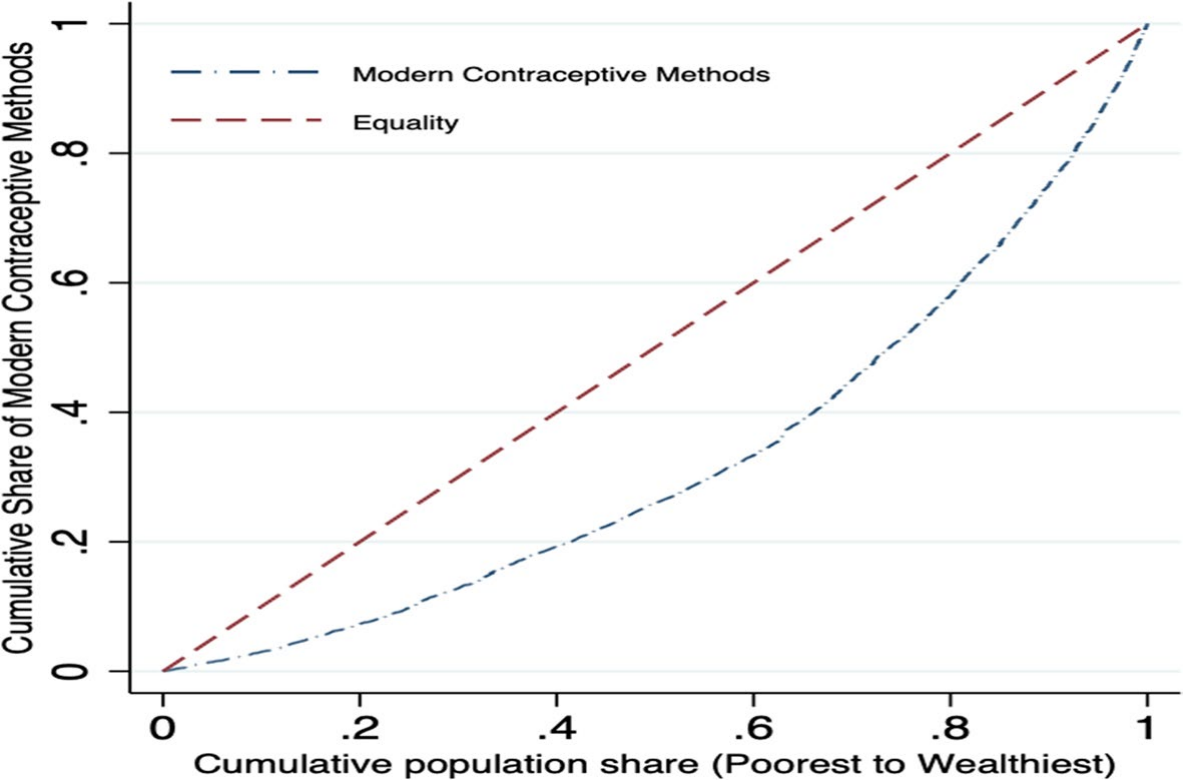
Concentration curve of the modern contraception use 2015-16 (NFHS-4), India

**Fig. 2:**
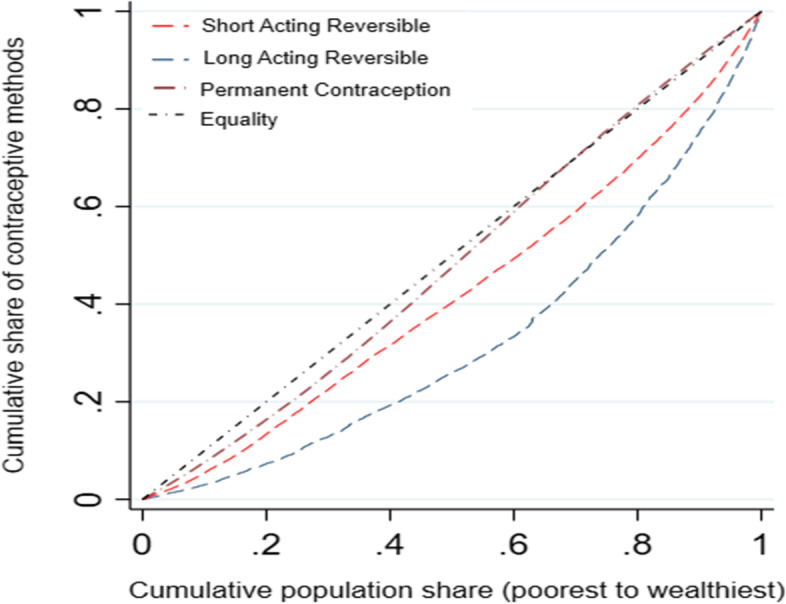
Concentration curve for the short-acting reversible, long acting reversible, and permanent contraception, 2015-16 (NFHS-4), India

Figures 1 and 2 employ concentration curves (CCs) to show that inequality in current contraceptive use favours the non-poor. With different modern contraceptive methods, the CCs constantly diverge from the line of inequality, implying a worsening of socioeconomic inequality with time. The increase in inequality was substantially greater among women from wealthier households than among women from poorer households.

There appears to be a magnitude difference between the curves. The requirement for a summary measure is critical considering this ambiguous picture. This requires the use of concentration indices, which are shown in Table 3.

**Table 3:** Concentration indices of different contraception methods, 2015-16 (NFHS-4), India

	**Short Acting Reversible(SARC)**	**Long Acting Reversible(LARC)**	**Permanent Contraception**	**Total**
Concentration index	0.0565***	0.020***	0.046***	0.122***
Std. error	0.002	0.001	0.004	0.004
N	499,627	499,627	499,627	499,627

Notes: Standard errors in parentheses; *p < 0.10, **p < 0.05, ***p < *0.01*

In Table 3, all concentration indices were positive and statistically different from zero indicating non-poor socioeconomic inequality in the various modern contraception methods, thereby supporting the results from the CCs in Fig. 2. Examining the trend, EI for different modern contraceptive methods changed from 0.06 (*p* < 0.01) in SARC methods to 0.02 (*p* < 0.01) in LARC methods and 0.05 (*p* < 0.01) in permanent contraceptives. Given the evidence of the existence of socioeconomic inequality in the different contraception methods, we decompose the CI to determine the contributing factors and how these explain the observed differences. But we only present the results showing the contribution of each of the determinants to observed socioeconomic inequality in the different modern contraceptive use.

**Fig. 3:**
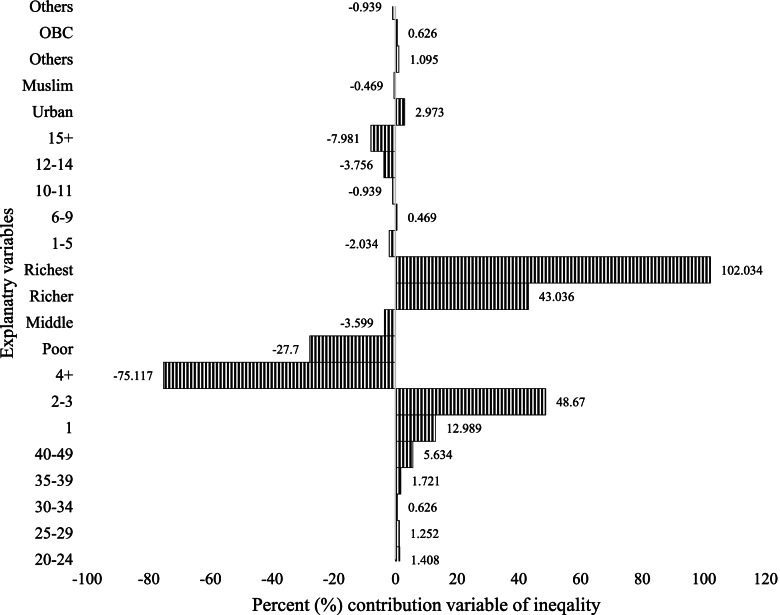
Decomposition of the concentration index for modern contraception methods, 2015-16 (NFHS-4), India

Figure 3 shows the decomposition results of the total modern contraception. The y-axis represents the percentage contribution to the socioeconomic inequality in modern contraceptive use of each variable in the regression model, whereas the x-axis indicates the variable of interest. The results in the tables show that women from the richest and richer households, women with 2–3 and 1 children, and women aged 40–49 years have a significant inequality in modern contraceptive use.

**Fig. 4:**
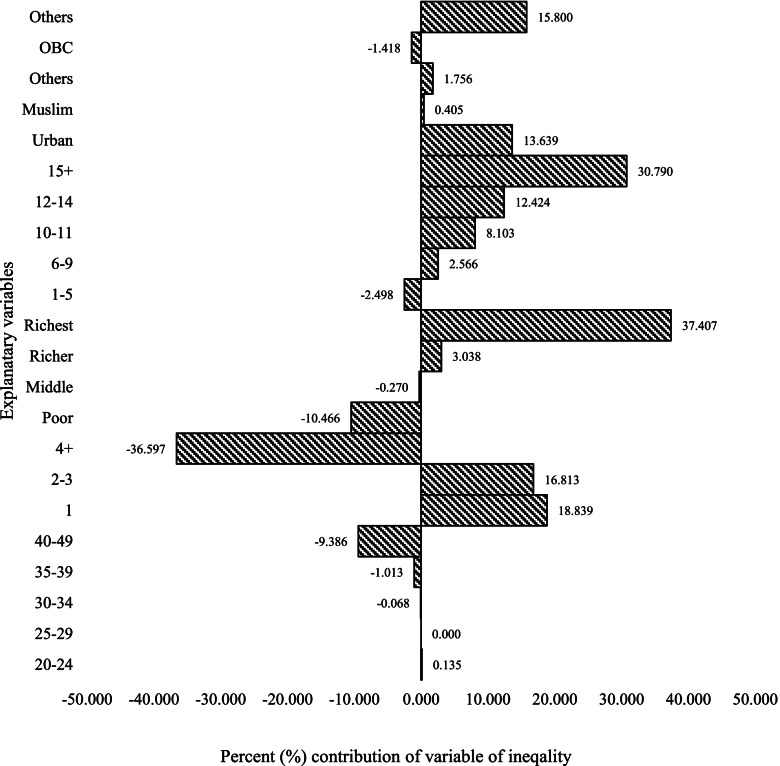
Decomposition of the concentration index for short-acting reversible methods, 2015-16 (NFHS-4), India

**Fig. 5:**
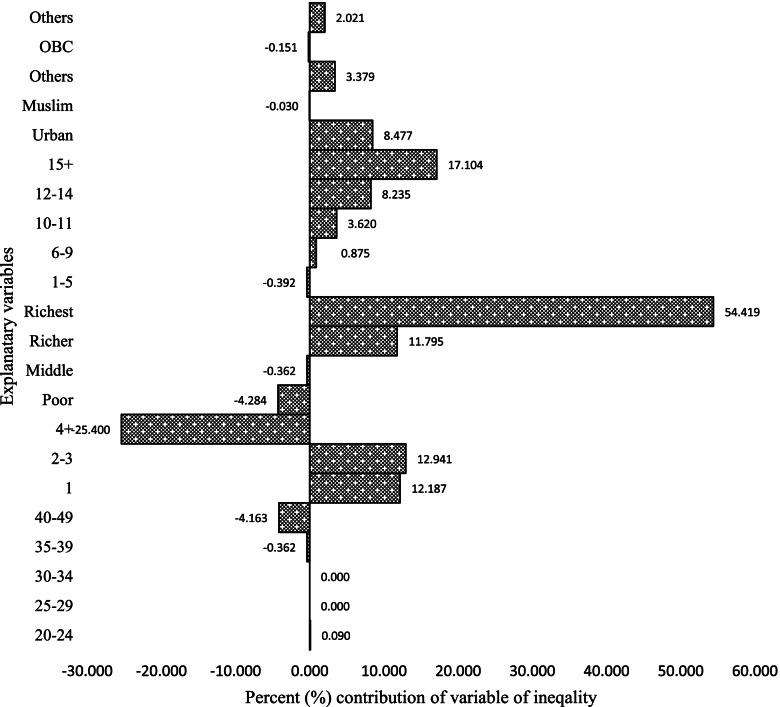
Decomposition of the concentration index for long-acting reversible methods, 2015-16 (NFHS-4), India

**Fig. 6:**
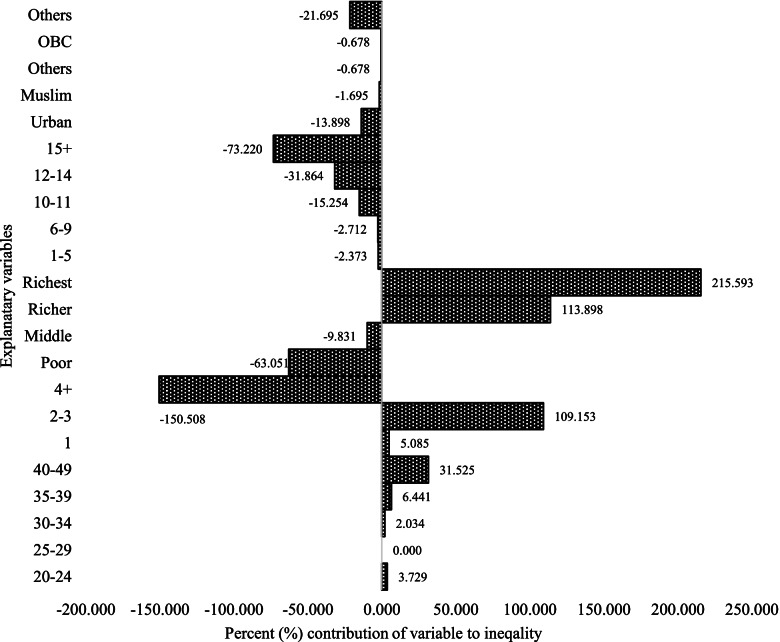
Decomposition of the concentration index for permanent contraception methods

In Figs. 4, 5, and 6, in different contraceptive methods, the women who have 15 + years of education contributed positively to the modern contraception methods, except for the negative contribution to the permanent contraception methods. It accounted for almost 31% of SARC methods (Fig. 4), 17% of LARC methods (Fig. 5), and contributed negatively to -73% of permanent contraception methods (Fig. 6). If we look at modern contraceptive use, women with 15 years of education have a negative contribution -8% (Fig. 3). The aggregate wealth-related inequality is 29.71% in SARC methods (Fig. 4), 61.56% in LARC methods (Fig. 5), and 257% in permanent method (Fig. 6) inequality of the aggregate wealth-related inequality, which suggests the existing wealth-related inequality among the richer population.

## Discussion

Using cross-sectional data from the fourth round of NFHS, the present study found that 32% of modern contraception users used permanent contraception, about 10% used short-acting reversible contraceptives (SARC), and only 1.9% used long-acting reversible contraceptive (LARC) methods. The findings were consistent with previous research, which revealed that the proportion of modern contraceptive users, which we classified into three different methods in our study, was lower in the younger reproductive age group, owing to early childbearing years and a higher proportion of currently married women in the same age group [2–4]. The current study found a lower percentage of SARC or LARC methods, which is still questionable, in contrast to previous studies demonstrating higher awareness of family planning methods and improved women's educational level [5]. Many factors that have been identified in many earlier studies, including lack of awareness, misinformation or persistent fear of side effects, and insufficient provider training, are likely to contribute to this small share which has become a potential barrier to uptake [6–9].

There were differences in the mix of modern contraception methods by sociodemographic characteristics such as educational attainment, place of residence, religion, caste, and wealth status [5, 10, 11]. However, permanent contraception methods have been found to have different patterns than the two remaining modern methods of contraception in the present study. For instance, women with higher education and who live in urban areas were more likely to use the LARC and SARC methods, although lower education and those who live in rural areas more frequently use the permanent contraceptive method. One possible explanation is that the continued dominance of sterilization in the Indian family planning program is mostly determined by socioeconomic factors, and thus women from more marginalized backgrounds rely on permanent contraception the most [5, 13, 25].

However, consistent findings documented large inequalities in modern contraceptive use among countries with higher economic inequalities [14]. Economic inequality in the use of modern contraception has decreased considerably as the uptake of modern contraception among women from lower socioeconomic backgrounds has increased [15]. Despite the widespread use of contraception, inequalities in modern contraceptive methods have long been a matter of concern in developing nations, including India [16]. The results of this study show that there is inequality in the use of three different types of modern contraceptive methods, which vary depending on associated socioeconomic factors. These inequalities are attributed to differences in the distribution of wealth index, education, caste, and place of residence.

The study found that the largest contributions to inequality in using SARC or LARC methods come from the educational attainment of women and their household wealth. However, the findings indicate that women’s education revealed greater inequalities for SARC methods than LARC, which is supported by previous research [17]. In agreement with our research, other studies also have found that women from low-income households and those who are uneducated have limited access to these contraceptive methods and are less aware of their benefits and efficacy [5, 17, 18]. A study conducted in Ethiopia indicated that the efficacy of short-acting methods among reproductive-aged women is mainly dependent on socioeconomic factors such as education and economic status [19]. One possible explanation for these findings could be the accessibility and affordability of these two modern contraceptive methods in India, particularly among low-income populations. Over the decades, the private sector in India has been recognized as a crucial function in providing family planning provisions, which might be considered an important factor in reducing contraception access among the poor [15, 20].

On the other hand, when it comes to permanent methods, the inequalities have shifted towards the older reproductive-aged women along with household wealth. India was the first country to start a family planning program, especially creating a department focused on sterilization [29]. Due to the limited availability of low-cost, high-efficacy methods, India has seen a higher uptake in permanent modern contraceptive methods, with a skewed preference for female sterilization [17]. This could be explained by the greater influence of socio-cultural norms on the method chosen, as well as a lack of awareness of alternative useful modern methods, which may lead them to view sterilization as a means of birth control. Parallel to these findings, a recent study has underlined the increased reliance on female sterilization in India over the last two decades, which has been linked to various characteristics, including poor household wealth, illiterate women, and lack of access to media [30].

Considering the age of the women, inequality for the use of permanent methods is higher than in SARC and LARC methods, especially female sterilization, because women over 40 years are more likely to desire a permanent form of contraception as a result of achieving the desired number of children. Declining marriage age, early childbearing age, lower contraception rates, and misconceptions about side effects among younger people have all contributed to the need for increased family planning programme awareness and promotion of useful and cost-effective modern contraceptives.

There are certain limitations to this study. The use of modern contraception by women is self-reported; nevertheless, this information may be subject to recall bias and social desirability bias, which can impact estimates. Because sterilization is so prevalent in Indian family planning policies, the findings may have limited generalizability beyond India. Because of the dominance of sterilization in Indian family planning policy, the results may have limited generalizability beyond India.

However, the study also presents some strengths. Firstly, we have mainly used modern methods of contraception, which are used by the majority of Indian women. Secondly is the evaluation of the inequalities according to the socioeconomic characteristics of women. This is important because identifying which subgroup of women is left behind will facilitate additional interventions to be targeted those who are most in need.

## Conclusions

This research adds to a growing body of studies directed at explaining the inequalities in modern contraceptive use based on socio-demographic variables. Women with more education and wealth are more likely to adopt LACR approaches rather than SARC methods. There is a need to make LARC and SARC methods more accessible, which will likely lead to a change away from target-based incentives and toward women-centred care. Simultaneously, there is a need to change the long-standing policy of adolescent marriage, childbearing soon after marriage, and early sterilization once the desired number and sex of children have been achieved. Male participation in family planning programmes should be prioritized to sustain established equity. Reduced unmet contraceptive needs and increased knowledge of contraception use among underprivileged women can help to narrow the inequality between LARC and SARC methods.

## Data Availability

The dataset used in this article is available only upon request on the DHS website at https://dhsprogram.com/what-wedo/survey-Types/dHs.cfm

## Footnotes

1 SARC includes pills, injectable, and condoms; while LARC includes intrauterine devices, implants; and Permanent contraception methods (i.e., male and female sterilization).
